# MARITAL STATUS AND VASCULAR EVENTS IN A DIVERSE NORTHERN MANHATTAN COHORT

**DOI:** 10.1093/geroni/igae098.4322

**Published:** 2024-12-31

**Authors:** Courtney Gao, Jackson Roberts, Tatjana Rundek, Mitchell Elkind, Hannah Gardener, Jose Gutierrez

**Affiliations:** Columbia Presbyterian Medical Center, New York, New York, United States; Massachusetts General Brigham, Boston, Massachusetts, United States; University of Miami, University of Miami Miller School of Medicine, Florida, United States; Columbia University Irving Medical Center, New York, New York, United States; Leonard M. Miller School of Medicine, University of Miami, Miami, Florida, United States; Columbia University, New York, New York, United States

## Abstract

**Background:**

Studies have shown that marital status is related to vascular outcomes but with potential variation by race/ethnicity. It remains unclear whether these associations occur in predominantly Hispanic populations.

**Methods:**

In the Northern Manhattan Study prospective cohort, we assessed participants ≥40 years and stroke-free at baseline. We categorized participants as married or unmarried, a group subdivided into divorced/separated, widowed, or single. We used adjusted hazard models to relate marital status to myocardial infarction (MI), stroke, vascular death, and all-cause death.

**Results:**

Of 2865 participants (53% Hispanic, mean 69 ±10 years), 32% were married, 16% were single, 28% were widowed, and 25% were divorced/separated. During a mean follow-up time of 15 years (IQR 3-28),14% had a stroke, 8% had an MI, and 68% died. Unmarried participants had a higher risk of any vascular event (aHR, 1.20 [95% CI, 1.04-1.39]) and all-cause death (aHR, 1.14 [95% CI, 1.02-1.28]) compared to married participants. The direction of the hazard ratios for stroke (aHR, 1.06 [95% CI, 0.83-1.35]) and MI (aHR, 1.11 [95% CI, 0.81-1.51]) was consistent with an increased risk. Divorced/separated participants had a higher risk of vascular events (aHR, 1.35 [95% CI, 1.14-1.61], vascular death (aHR, 1.29 [95% CI, 1.05-1.59]), and all-cause death (aHR, 1.21 [1.06-1.39]) compared to married participants.

**Conclusion:**

In a predominantly Hispanic cohort, unmarried participants had a higher risk of vascular events and all-cause death. Future studies should explore the impact of risk stratification of patients based on marital status and provision of support to patients undergoing marital transition.

